# Supplementary material for: Aberrant Methylation of SLIT2 Gene in Plasma Cell-Free DNA of Non-Small Cell Lung Cancer Patients
Source: Cancers (Basel). 2022 Jan 7;14(2):296. doi: 10.3390/cancers14020296 (PMC8773699; doi:10.3390/cancers14020296)
Supplement: Supplementary file 1 [file cancers-14-00296-s001.zip › cancers-1486796-supplementary.pdf]

Supplementary Material: Aberrant Methylation of SLIT2 Gene in Plasma Cell-Free DNA of Non-Small Cell Lung Cancer Patients

Yujin Kim, Bo Bin Lee, Dongho Kim, Sang-Won Um, Joungho Han, Young Mog Shim and Duk-Hwan Kim

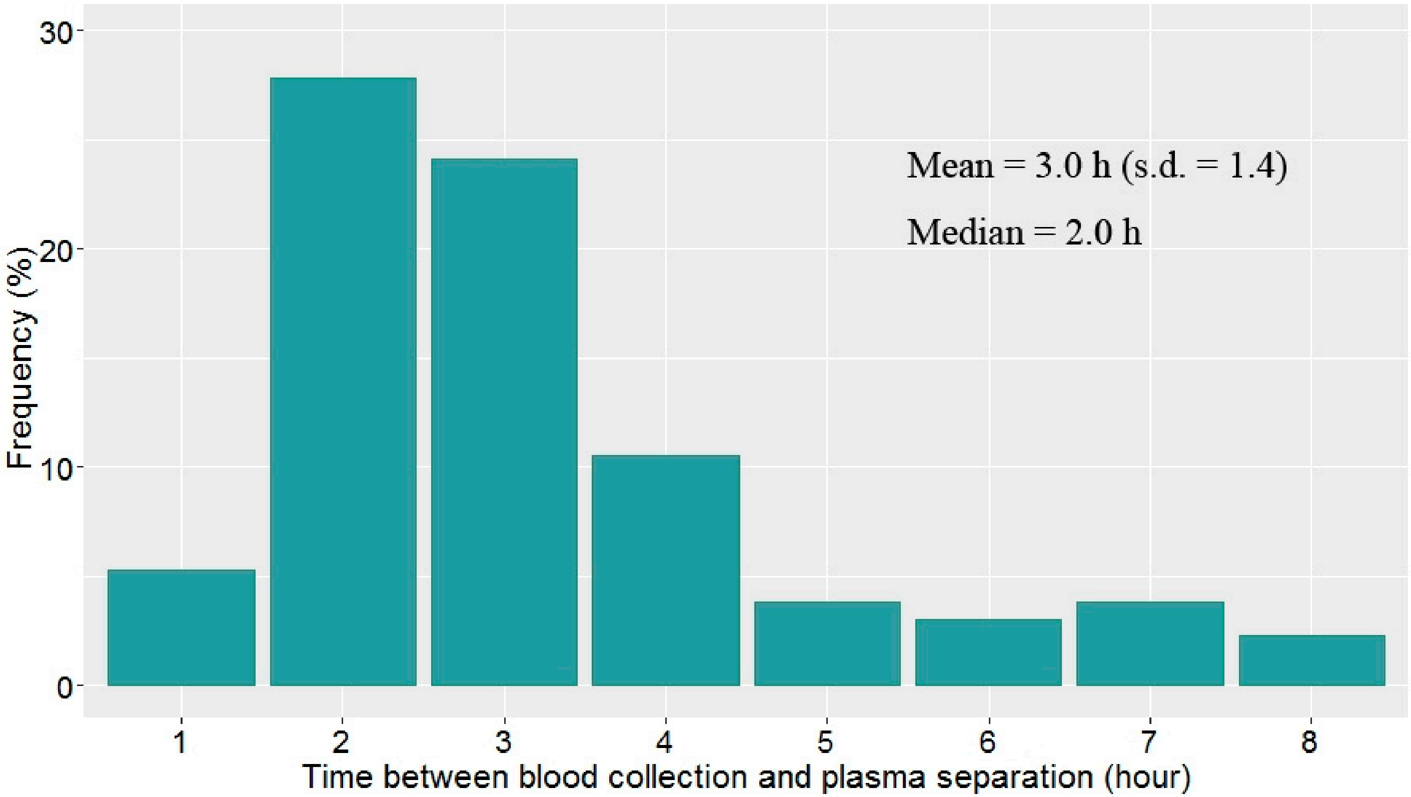

Figure S1. Distribution of plasma separation time after blood collection. The distribution of time from blood collection to plasma separation was analyzed in 133 patients. "h" and "s.d" represent hour and standard deviation.

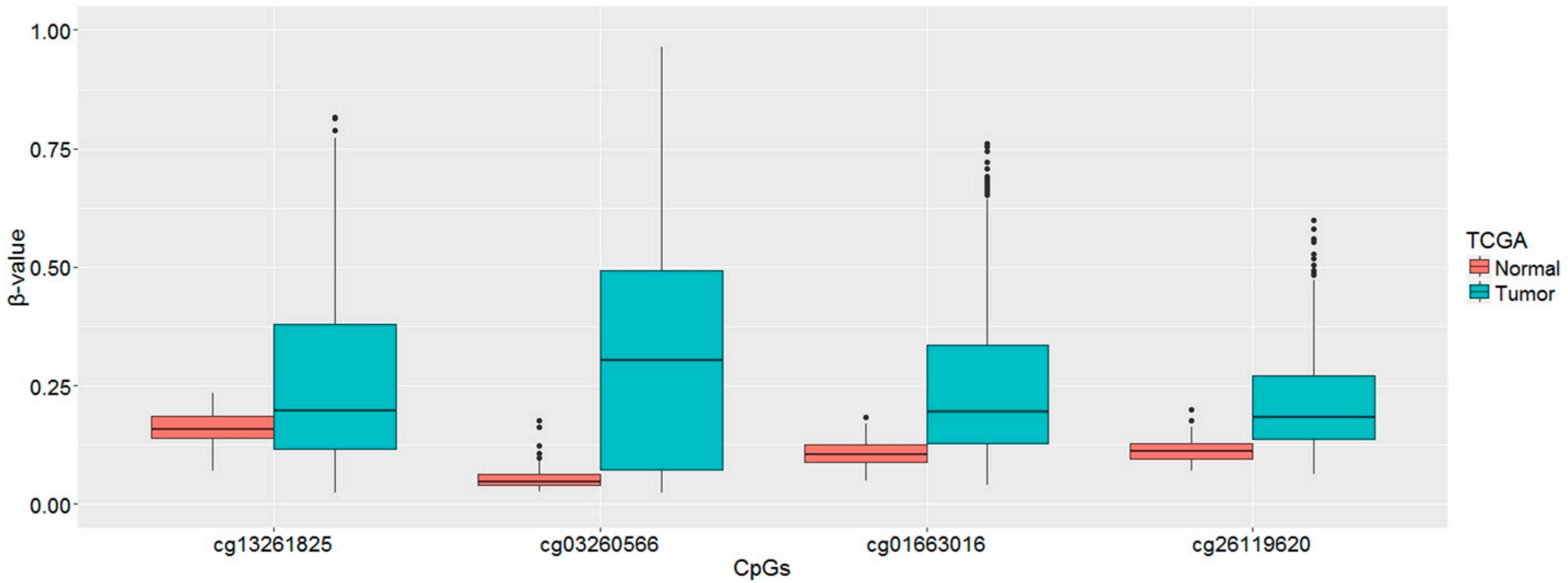

Figure S2. Aberrant methylation of *SLIT* genes in TCGA lung cancer cohort. Methylation levels of five CpGs showing statistical significance in our data were compared between normal (*N* = 74) and tumor (N=824) tissues in the TCGA lung cancer dataset. The cg14226472 in *SLIT1* gene was not compared because there was no data in the TCGA dataset. Y axis represents β-values.

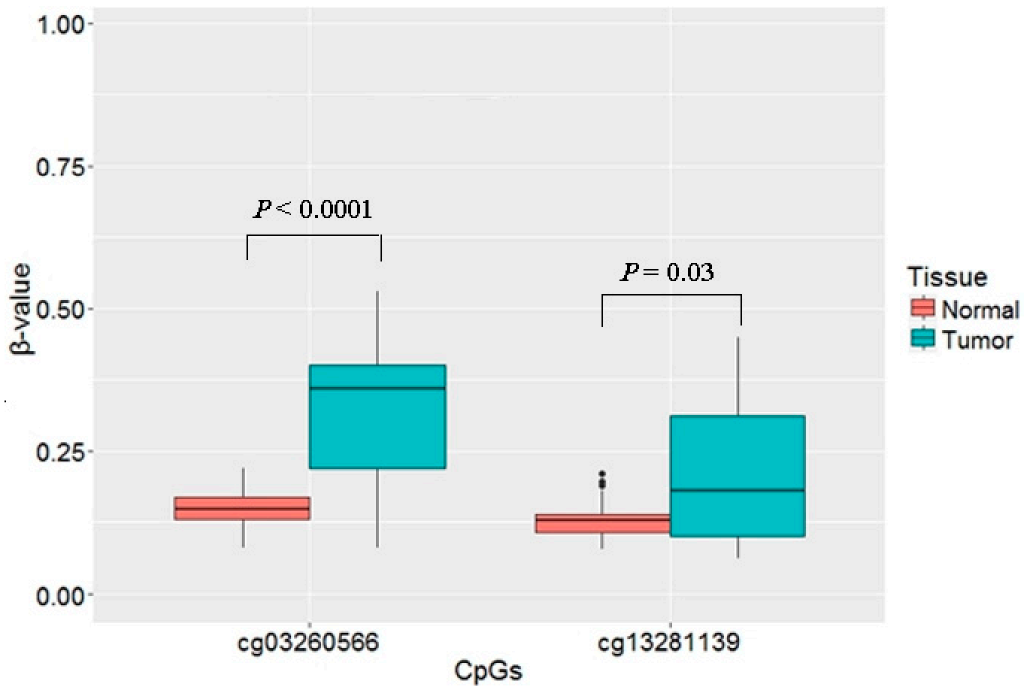

Figure S3. Degree of methylation of cg03260566 and cg13281139 in NSCLC. Methylation levels of cg03260566 and cg13281139 were compared between tumor and matched normal tissues from 42 NSCLC patients. *P*-values are based on Wilcoxon rank sum test. Y axis represents β-values.

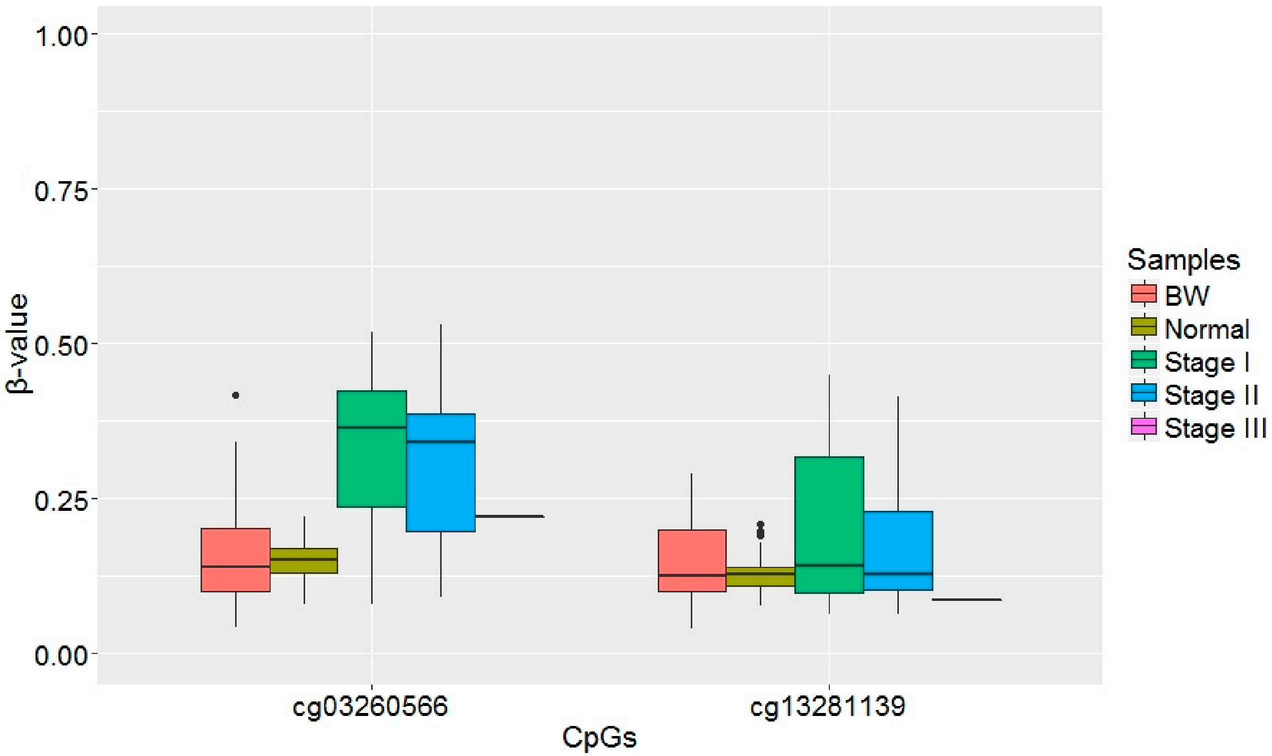

Figure S4. Methylation levels of cg03250566 and cg1328119 according to pathologic stage. Methylation levels of cg03260566 and cg13281139 were compared in bronchial washing (BW) samples from 60 healthy individuals, matched normal tissues (Normal) from 42 42 NSCLC patients, and tumor tissues from stage I-III NSCLC. Y axis represents β-values.

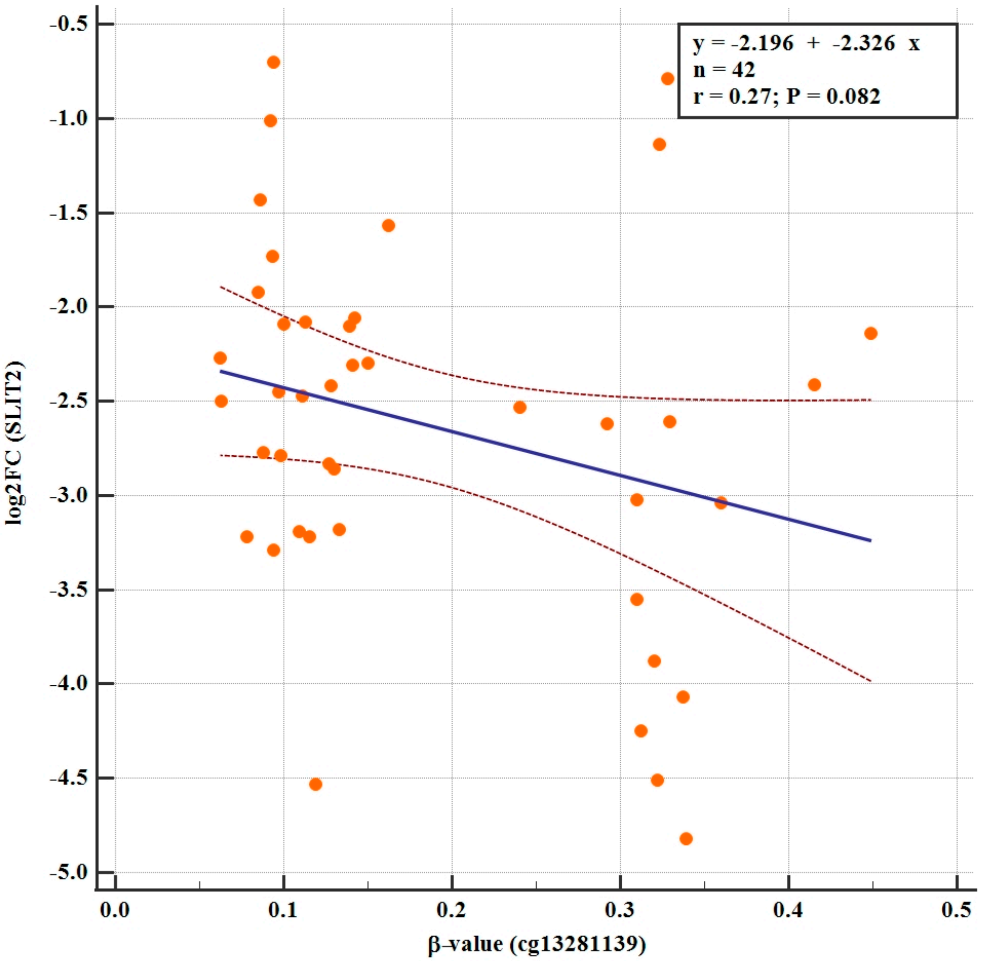

Figure S5. Correlation between cg13281139 methylation and SLIT2 mRNA levels. Correlation between methylation levels of cg13281139 and log2 fold change (tumor/normal) of SLIT2 mRNA was analyzed. The plot was constructed using the MedCalc (version 19.0.5) software. *P*-value is based on Spearman's rank correlation coefficient.

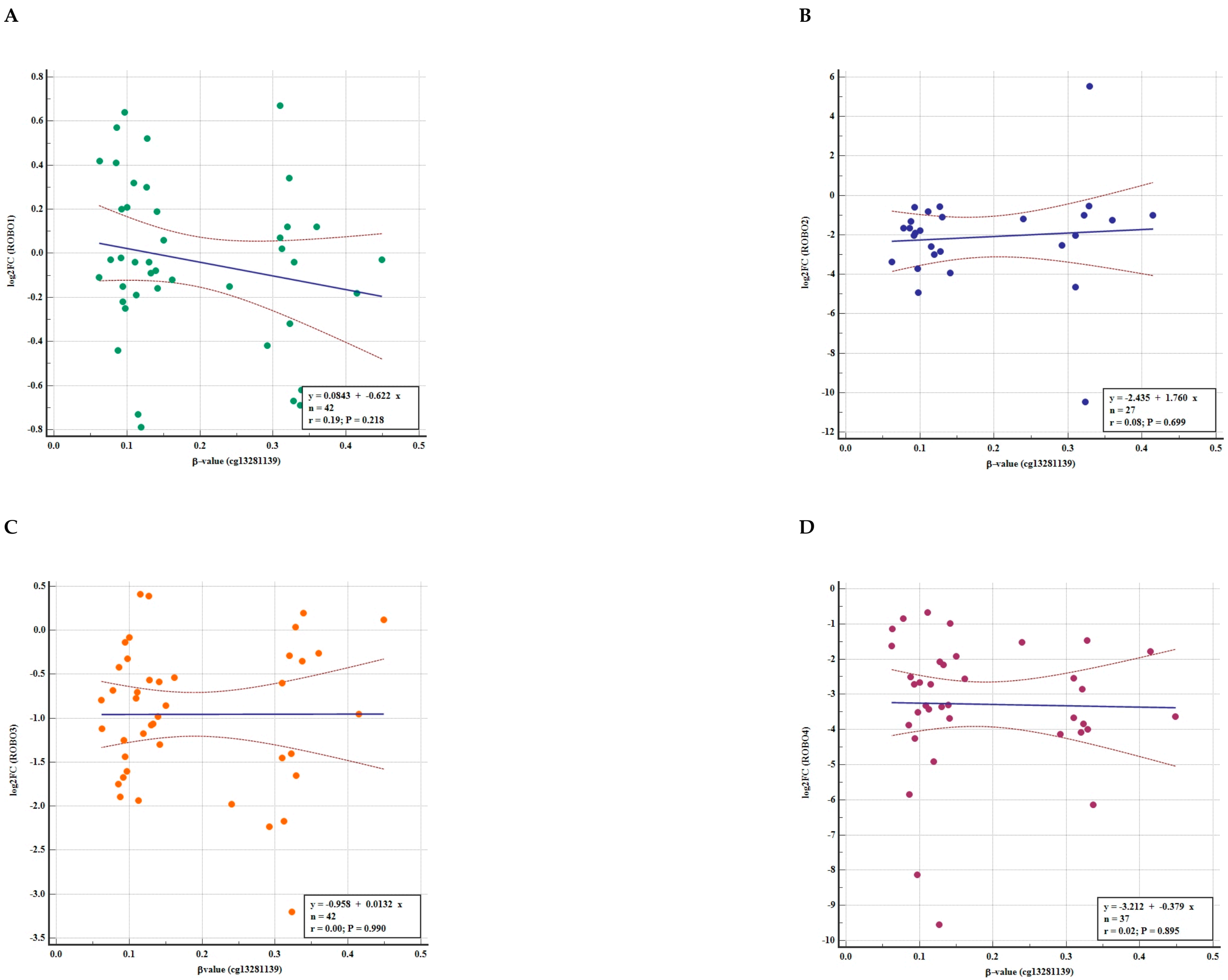

Figure S6. Relationship between cg13281139 methylation and ROBOs expression. Correlation between methylation levels of cg13281139 and log2 fold change (tumor/normal) for mRNA levels of ROBO1 (A), ROBO2 (B), ROBO3 (C) and ROBO4 (D) was analyzed in 42 NSCLCs. The scatter plots were created using the version 19.0.5 of MedCalc software. *P*-values are based on Spearman's rank correlation coefficient.

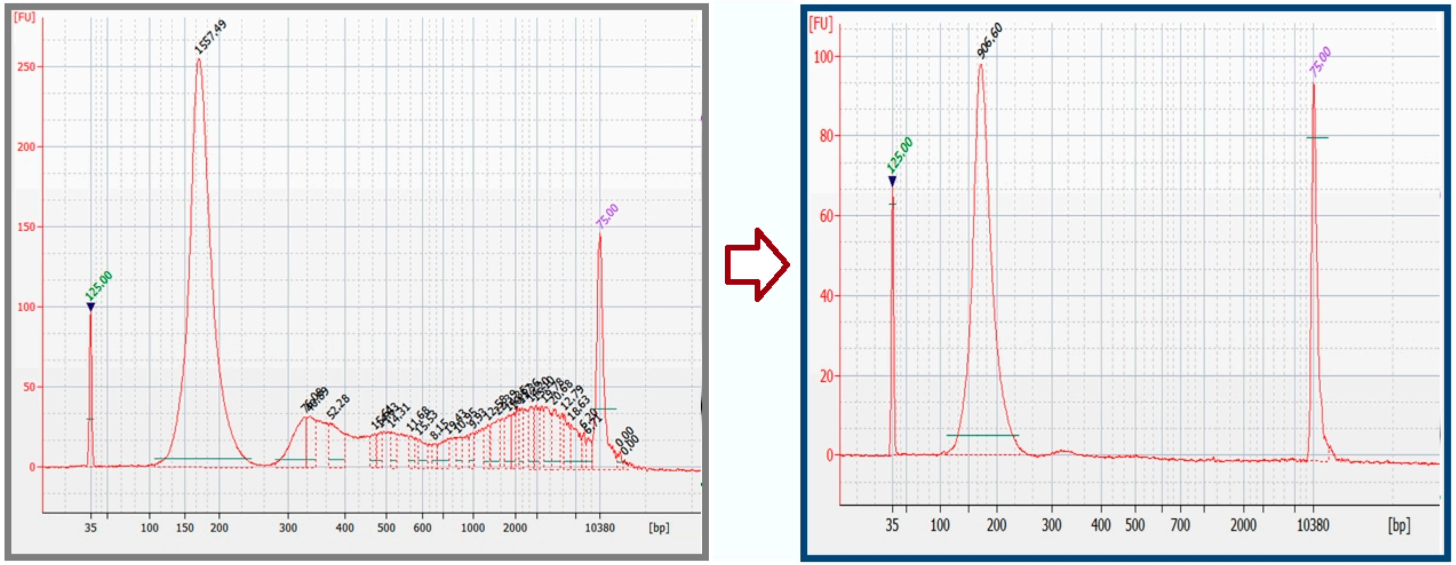

Before **After**

Figure S7. Electropherogram after clearance of genomic DNA with 2% isopropanol.

Table S1. Primer sequences for pyrosequencing.

Primers	Sequences
Forward	5′-GTTGGGGTTTTTAAYGGTTGTTAAGATGTA-3′
Reverse	5′-[Biotin]ATACCTATTAAAATCCCCTCTTCTAT-3′
Sequencing	5′-GTTTTTAAYGGTTGTTAAGATGTAG-3′
Sequence to analyze	GGGTYGGTTG TTGTGGGGAG GGYGGTTTTT TGATTTTA

Table S2. CpGs information**.**

TargetID	UCSC_REFGENE_NAME	UCSC_CPG_ISLANDS_NAME	UCSC_REFGENE_GROUP	RELATION_TO_UCSC_CPG_ISLAND	MAPINFO
cg01663016	SLIT3	chr5:168727429-168728275	5'UTR;1stExon	Island	168727752
cg03260566	SLIT2	chr4:20253276-20256868	Body	Island	20256841

cg13261825	SLIT1	-	Body	-	98939742
cg14226472	SLIT1	chr10:98945062-98946239	TSS1500	Island	98946111
cg26119620	SLIT3	chr5:168727429-168728275	Body	N_Shore	168727088

Abbreviations: UCSC_REFGENE_NAME, Gene name (UCSC); UCSC_CPG_ISLANDS_NAME, CpG island name (UCSC); UCSC_REFGENE_GROUP, Gene region feature category (UCSC); Relation_to_UCSC_CpG_Island, Relationship to Canonical CpG Island, Shores - 0-2 kb from CpG island; Shelves - 2-4 kb from CpG island. MAPINFO, Coordinates - genome build 37.

Table S3. Relationship between *SLIT2* hypermethylation and clinicopathological variables (*N* = 42).

**Variables**	**Hypermethylation of cg03260566**		***P*-value**	**Hypermethylation of cg13281139**		***P*-value**
	**No (*N* = 15)**	**Yes (*N* = 27)**		**No (*N* = 29)**	**Yes (*N* = 13)**	
Age	64 ± 5	66 ± 6	0.70	65 ± 10	69 ± 8	0.37
Tumor size (cm)	5.1 ± 2.1	4.3 ± 1.3	0.23	4.5 ± 1.6	4.8 ± 1.7	0.44
Pack-years	32 ± 30	26 ± 26	0.47	25 ± 29	40 ± 16	0.09
Sex						
Men	9	18		17	10	
Women	6	9	0.67	12	3	0.31
Histology						
Adeno	8	19		20	7	
Squamous	4	4		3	5	
Others	3	4	0.58	6	1	0.13
Pathologic stage						
I	8	18		17	9	
II	6	9		11	4	
III	1	0	0.38	1	0	0.82
IV						
Lymphatic invasion						
No	7	12		11	8	
Yes	8	15	0.89	18	4	0.17
Neural invasion						
No	12	22		27	11	
Yes	3	5	0.91	2	2	0.58
Vascular invasion						
No	13	25		22	12	
Yes	2	2	0.61	7	1	0.40

*abbreviations: Adeno, adenocarcinoma; Squamous, squamous cell carcinoma.

Table S4. Clinicopathological characteristics.

Variables	Cancer (*N* = 72)	Cancer-free (*N* = 61)
Age	61 ± 10	54 ± 11
Sex		
Male	54	46
Female	18	15
Histology		
Adeno	31	
Squamous	30	
Others	11	
Pathologic stage		
I	41	
II	26	
III	3	
IV	2	

Abbreviations: Adeno, adenocarcinoma; Squamous, squamous cell carcinoma.

Table S5. mRNA fodl change of SLIT2 and ROBOs in 42 NSCLCs.

Variable	Log2FC[a]	Paired *t*-test
SLIT2	-2.68	2.40E-21
ROBO1	0.17	0.33
ROBO2	-2.40	3.34E-06
ROBO3	-0.95	1.03E-09
ROBO4	-3.28	1.02E-12

[a] FC indicate fold change of mRNA levels in cancer vs. normal tissues.

Publisher's Note: MDPI stays neutral with regard to jurisdictional claims in published maps and institutional affiliations.

© 2022 by the authors. Licensee MDPI, Basel, Switzerland. This article is an open access article distributed under the terms and conditions of the Creative Commons Attribution (CC BY) license (http://creativecommons.org/licenses/by/4.0/).
